# Six-month quality-of-life and functional status of acute respiratory distress syndrome survivors compared to patients at risk: a population-based study

**DOI:** 10.1186/s13054-015-1062-y

**Published:** 2015-10-02

**Authors:** Michelle Biehl, Rahul Kashyap, Adil H. Ahmed, Martin K. Reriani, Uchenna R. Ofoma, Gregory A. Wilson, Guangxi Li, Michael Malinchoc, Jeff A. Sloan, Ognjen Gajic

**Affiliations:** Multidisciplinary Epidemiology and Translational Research in Intensive Care (METRIC) Group, Mayo Clinic, Rochester, MN USA; Department of Internal Medicine, Division of Pulmonary and Critical Care Medicine, Mayo Clinic, Rochester, MN USA; Sanford USD Medical Center, 1205 S. Grange Avenue, Suite 407, Sioux Falls, SD USA; Wichita Falls Family Practice Residency Program (WFFRP), North Central Texas Medical Foundation, Wichita Falls, TX USA; Department of Critical Care Medicine, Geisinger Medical Center, Danville, PA USA; Department of Medicine, Guang An Men Hospital, China Academy of Chinese Medical Science, Beijing, China; Health Sciences Research, Division of Biomedical Statistics and Informatics, Mayo Clinic, Rochester, MN USA

## Abstract

**Introduction:**

The long-term attributable burden related to acute respiratory distress syndrome (ARDS) is not fully investigated. The aim of this study is to evaluate the quality of life (QOL) and functional status at 6 months after hospitalization in patients at risk for ARDS who did and did not develop the syndrome.

**Method:**

This is a population-based prospective cohort study of adult patients from Olmsted County, Minnesota, with or at risk for ARDS hospitalized from October 2008 to July 2011. The primary outcomes were changes in QOL and functional status, measured through 12-Item Short Form Survey (SF-12) and Barthel Index (BI) respectively, from baseline to 6 months, compared between survivors who did and did not develop ARDS.

**Results:**

Of 410 patients with or at risk for ARDS, 98 had baseline surveys collected and 67 responded to a 6-month survey (26 ARDS, 41 non-ARDS). Both ARDS and non-ARDS groups had lower physical component of SF-12 at baseline compared to general population (*P* < 0.001 for both). ARDS patients had poorer baseline functional status compared to non-ARDS (mean BI 80 ± 25 vs. 88 ± 22, *P* = 0.03). No significant differences were observed for the change between 6 months and baseline BI (delta 2.3 for ARDS vs. 2.0 for non-ARDS, *P* = 0.5), or mental (delta 2.7 vs. 2.4, *P* = 0.9) or physical (delta –3 vs. –3.3, *P* = 0.9) component of SF-12 between survivors with and without ARDS.

**Conclusion:**

In this population-based study, decreased QOL and functional status 6 months after hospitalization were largely explained by baseline condition, with similar recovery in survivors who did and did not develop ARDS.

**Electronic supplementary material:**

The online version of this article (doi:10.1186/s13054-015-1062-y) contains supplementary material, which is available to authorized users.

## Introduction

Acute respiratory distress syndrome (ARDS) commonly complicates acute illness in the intensive care unit (ICU). This syndrome is associated with high morbidity and mortality and tremendous costs [1]. With advances in treatment and supportive measures [2, 3], an increasing number of patients are surviving the syndrome with remarkable lung recovery. However, outcome studies show that many ARDS survivors develop long-term neurocognitive, neuropsychological, neuromuscular, functional and quality of life (QOL) impairment [4–13].

While many studies show both short- and long-term decrease in QOL among ARDS survivors when compared to the sex- and age-matched general population [6, 8–10], the amount of decline directly attributable to ARDS remains unclear. For the purpose of prioritizing future prevention strategies, it is imperative to study the attributed burden of this condition by quantifying specific patient-centered outcomes. In addition, data on long-term QOL and functional status of ARDS survivors has traditionally come from a secondary analysis of selected clinical trial populations, which may involve selection bias, as a very small proportion of eligible patients are usually enrolled in randomized clinical trials. Additionally, there are limited data on long-term outcomes of ARDS survivors with the assessment of their pre-morbid condition [12, 14].

To evaluate the attributed burden of ARDS we evaluated the QOL and functional status at 6 months post hospitalization compared to baseline in a population-based cohort of patients with ARDS, or at high risk of ARDS.

## Methods

This is a prospective, population-based cohort study. It was approved by the Mayo Clinic Institutional Review Board (IRB # 08-003560) and informed consent was obtained from all eligible patients.

### Study population

The study protocol has been published previously [15]. Eligible patients included adult residents from Olmsted County, Minnesota, who were admitted to the Mayo Clinic between October 2008 and July 2011 with one or more ARDS predisposing conditions (sepsis, pneumonia, aspiration, pancreatitis, shock, high-risk trauma, and high-risk surgery) within 6 hours of hospital admission [16]. We excluded the following patients: those admitted for cardiac telemetry, coronary care, low-risk elective surgeries, or labor and delivery; patients age <18 years; patients who were treated for comfort care or hospice care only; patients who had ARDS or pulmonary edema at the time of hospital admission; patients readmitted to the hospital; patients transferred from other hospitals; and patients who denied research authorization.

### Identification of patients at risk of developing ARDS

To identify patients at risk of developing ARDS for inclusion in the study, we used a previously validated acute lung injury (ALI) prediction model: the lung injury prediction score (LIPS) [17]. The LIPS model incorporates demographic, environmental, and clinical characteristics at the time of hospital admission to identify patients at high risk of ARDS [16].

### Ascertainment of development of ARDS

Screening for ARDS development was performed by previously validated electronic surveillance (ARDS “sniffer”) [18]. The electronic alert is triggered by the following combination of observations: 1) qualifying arterial blood gas analysis: the ratio of partial pressure of oxygen to inspired oxygen concentration (PaO_2_/FIO_2_) <200 and 2) qualifying chest radiograph report (free-text Boolean query containing trigger words: “bilateral” and “infiltrate” or “edema”). The ARDS sniffer demonstrated excellent negative predictive value at the time of hospital admission [18]. Trained investigators subsequently reviewed all ARDS sniffer alerts and confirmed the presence or absence of ARDS according to the standard definition based on the American-European consensus conference [19]. This process showed excellent inter-rater reliability [20].

### Severity of illness and comorbidities

Charlson comorbidity index at the time of hospital admission was calculated in all patients. APACHE III scores were calculated at the time of ICU admission.

### Quality of life and functional status

QOL was assessed using the 12-Item Short Form Survey (SF-12), which is a validated tool developed to replicate the 36-Item Short Form Survey (SF-36) and minimize respondent burden [21, 22]. Functional status was assessed using the Barthel Index (BI), one of the best measurement scales for activities of daily living, previously used in ICU survivors [23, 24]. These surveys were administered by trained study coordinators [21]. Assessments were performed at baseline and at 6 months from the first questionnaire date. The baseline survey was done during hospitalization; however, the patients were asked about their pre-morbid condition before acute illness. After informed consent was obtained, study coordinators decided whether the patient was competent enough to independently complete the questionnaire by performing the mini-mental status examination (MMSE). If the patient was deemed incompetent (abnormal MMSE) or too ill to complete the survey (unable to speak due to critical illness), a surrogate was acknowledged to help answer the questions on behalf of the patient. Follow-up contact information was attained, and the patients or their surrogates who successfully completed the baseline survey were contacted by the center personnel by telephone 6 months after that hospitalization. The pre-specified primary endpoint was the change at 6 months from baseline in QOL and functional status. QOL and functional status measures were then compared between patients who did and did not develop ARDS.

### Statistical analysis

The SF-12 was scored according to the normative standards established by Ware et al. [21], such that persons with a normal health-related quality of life (HRQOL) would have an average SF-12 score of 50, with a standard deviation (SD) of 10. Scores <50 indicate a poor HRQOL, while scores >50 indicate a better HRQOL. The SF-12 is standardized to the US population and it is described in means and SD. Categorical variables are presented as counts and percentages. Continuous data are summarized as means (SD) or medians (25 − 75 % interquartile range (IQR)). The paired *t* test was used to assess change (delta) in patients’ SF-12 scores between baseline and 6-month follow up (within patient change). The independent *t* test was used to compare the mean baseline, 6-month, and delta (6 months – baseline) SF-12 scores between ARDS and non-ARDS groups. The Wilcoxon signed rank test was used to assess change (delta) in patients’ BI scores between baseline and 6-month follow up (within patient change); and the Wilcoxon rank sum test was used to compare the BI in the two groups. Non-parametric tests were used for analysis of BI scores, because the data distribution was skewed. Butterfly (stream) plots were used to illustrate the trajectory of each patient’s functional status and QOL from baseline to 6 months.

A comparison of QOL was made between our study population and the general US population. The *t* test was used to evaluate whether our population mean SF-12 score was different from 50, which is the population norm [25]. Analysis of covariance was performed to evaluate ARDS as a predictor of the main outcomes (SF-12 and BI scores), after adjusting for age, sex and baseline values of SF-12 and BI scores. Additional sensitivity analysis was performed to address the survival bias where enrolled patients who died before their 6 months assessment were given the lowest possible scores (zero) for SF-12 and BI. A sensitivity analysis limited to self-answered surveys was used to address the potential bias, given the different percentage in survey response by the patients. The traditional *p* value cutoff ≤0.05 was used for statistical significance. Statistical analyses were performed using SAS (version 9.1, SAS Institute, Cary, NC, USA). Statistical graphs were developed using the R statistical software functions [26].

## Results

**Fig. 1:**
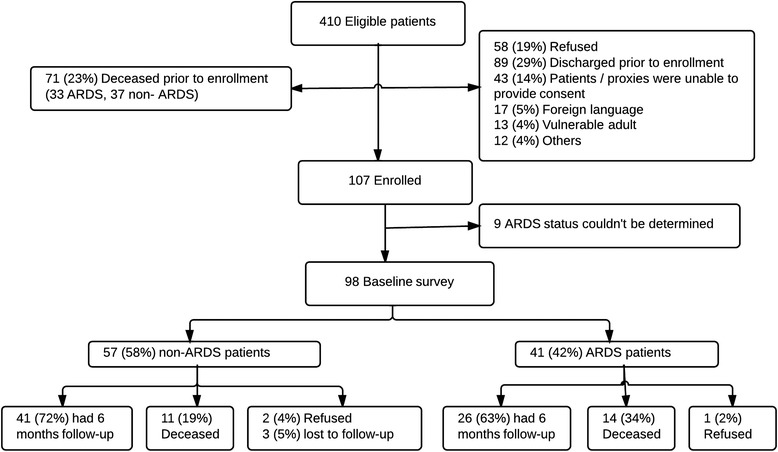
Consolidated Standards of Reporting Trials (CONSORT) flowchart diagram. *ARDS* acute respiratory distress syndrome

Of 410 patients who were identified with ARDS or at risk of ARDS at the time of hospital admission during the 3-year study period (2008 − 2011), 98 had baseline surveys collected (Consolidated Standards of Reporting Trials (CONSORT) flowchart diagram, Fig. 1). Eligible patients who did and did not complete the baseline survey had similar sex, race, ARDS status and Charlson comorbidity index (Additional file 1: Online Resource 1). Patients who did not complete the baseline survey were older (67 vs. 62 years), had a higher APACHE III score (80 vs. 71), were more often treated in the ICU (68 vs. 54 %), and had higher mortality (Additional file 1: Online Resource 1).

Of the 98 enrolled patients, 25 died, 3 refused to continue in the study, 3 were lost to follow up, and 67 survivors (92 % of those eligible) responded to a 6-month follow-up survey (26 patients with ARDS and 41 without) (Fig. 1). Baseline characteristics were similar between the groups with and without ARDS (Table 1). The ARDS patients had more days of invasive mechanical ventilation compared to non-ARDS patients, and longer ICU and hospital length of stay (Table 1).

**Table 1:** Characteristics of 6-month survivors with and without ARDS

	ARDS (n = 26)	Non-ARDS (*n* = 41)	*P* value
Age, median (IQR)	60.6 (50.6, 69.0)	59.4 (44.0, 70.9)	0.9^a^
Female sex, number (%)	13 (50)	20 (49)	0.9^b^
White race, number (%)	18 (69)	33 (80)	0.4^b^
ICU admission, number (%)	26 (100)	27 (66)	0.1^a^
Apache III 24 hours, median (IQR)	68.5 (53, 91.3)	71 (51, 79)	0.7^a^
Invasive mechanical ventilation, number (%)	23 (88)	14 (34)	<0.001^b^
Invasive MV days, median (IQR)	3.0 (0.3, 11.0)	0 (0, 2.0)	0.005^a^
ICU LOS, median (IQR)	7.9 (4.1, 18.1)	3.1 (1.1, 7.2)	0.005^a^
Hospital LOS, median (IQR)	19.5 (11.1, 32.6)	10.6 (6.1, 14.1)	0.004^a^
Self-answered questionnaires at 6-month follow up, number (%)	22 (85)	34 (83)	0.86^b^

^a^Wilcoxon rank sum test; ^b^Chi square test. *ARDS* acute respiratory distress syndrome; *LIPS* lung injury prediction score, *APACHE* acute physiology and chronic health evaluation; *MV* mechanical ventilation, *LOS* length of stay

Baseline surveys were self-answered by 75 % of the patients in the non-ARDS group, and by 54 % of the patients in the ARDS group *(p* = 0.02*).* Six-month surveys were self-answered by 83 % of the patients in the non-ARDS group, and by 85 % of the patients in the ARDS group *(p = 0.86).*

### Quality of life

#### Mental component score of SF-12

**Fig. 2:**
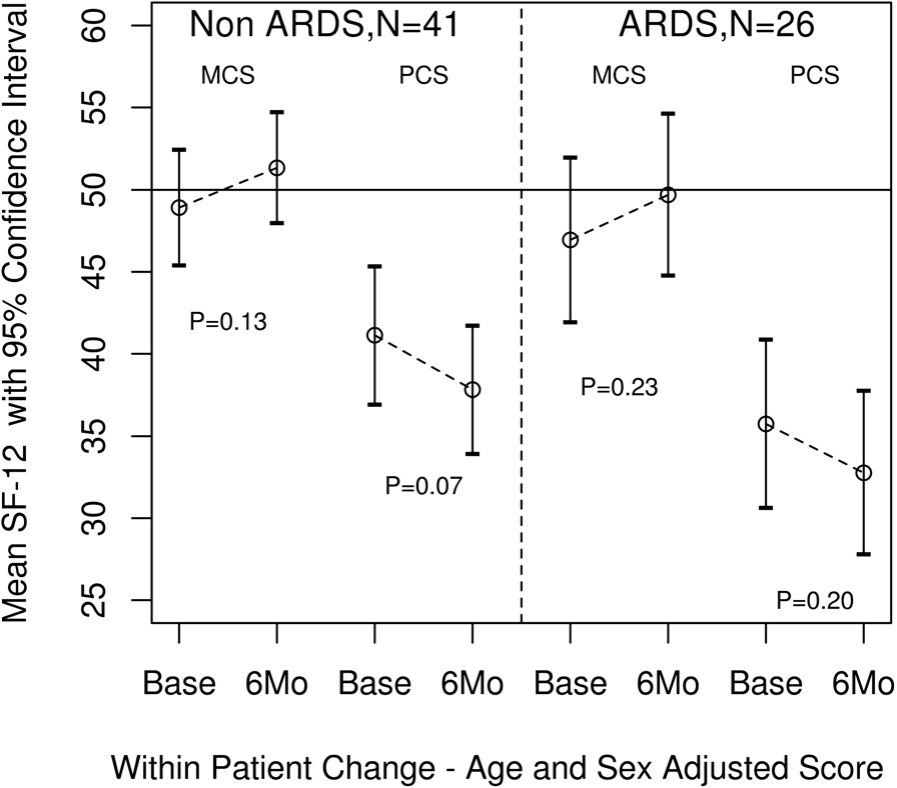
Mental and physical component scores for the 12-item Short Form Survey (SF-12) measured at baseline and 6 months in patients with acute respiratory distress syndrome (*ARDS*) and patients without ARDS (*non-ARDS*). *MCS* mental component score, *PCS* physical component score

There was no within-patient difference in the mental component score 6 months post baseline, either in the ARDS (49.7 at baseline vs. 46.9 at 6 months) or in the non-ARDS groups (51.3 vs. 48.9) (Table 2). There was also no difference in the mental component of SF-12 at baseline or at 6 months between patients with and without ARDS. Furthermore, there was no difference when we compared the change in mental component score (delta 6 months – baseline) between the two groups (Table 2 and Fig. 2). Overall the baseline mental component scores of SF-12 in both groups were comparable to the healthy population (SF-12 score of 50).

**Table 2:** Mental and physical component score of SF-12 at baseline and 6 months among patients with and without ARDS

	ARDS (n = 26)	Non-ARDS (n = 41)	*P* value for between-patient comparison^a^
SF-12 MCS baseline - mean ± SD	46.9 ± 12.4	48.9 ± 11.2	0.50
SF-12 MCS 6 months - mean ± SD	49.7 ± 12.2	51.3 ± 10.7	0.56
SF-12 MCS delta (difference in means)	2.7 (95 % CI −5.8, 5.2)	2.4 (95 % CI −5.6, 5.0)	0.9
P value for within-patient comparison^b^	0.23	0.13	
SF-12 PCS baseline - mean ± SD	35.7 ± 12.7	41.1 ± 13.3	0.11
SF-12 PCS 6 months - mean ± SD	32.8 ± 12.3	37.8 ± 12.3	0.11
SF-12 PCS delta (difference in means)	−3 (95 % CI −6.1, 5.5)	−3.3 (95 % CI −6.1, 5.4)	0.9
*P* value for within-patient comparison^b^	0.2	0.07	

^a^Independent *t* test; ^b^paired *t* test. *ARDS* acute respiratory distress syndrome, *MCS* mental component score, *PCS* physical component score, *SF-12* 12-Item Short Form Survey

#### Physical component score of the SF-12

Both the ARDS and non-ARDS groups had a lower physical component score of the SF-12 at baseline and at 6 months compared to the mean score of the general US population (ARDS group vs. general population at baseline, *p* <0.0001, and at 6 months, *p* <0.0001; non-ARDS group vs. general population at baseline, *p* = 0.0001, and at 6 months, *p* <0.0001).

As with the mental health score of the SF-12, there were no within-patient differences in either the ARDS or the non-ARDS group in the physical component score 6 months from baseline (ARDS group 32.8 at baseline vs. 35.7 at 6 months; non-ARDS group 37.8 vs. 41.1) (Table 2). There was also no difference at baseline or at 6 months in the physical component score between the ARDS and non-ARDS groups. Additionally, no difference was elicited when we compared the change in the physical component score (delta 6 months – baseline) between the two groups (Table 2 and Fig. 2).

### Functional status

The ARDS group had a significantly lower mean BI score at baseline and at 6 months compared to the non-ARDS group (Table 3). These scores indicate that ARDS patients had a deficit in their activities of daily living even before hospital admission. There was no within-patient change in BI scores at 6 months compared to baseline in either group. No difference was found in the change in BI score (delta 6 months – baseline) between the two groups.

**Table 3:** BI scores at baseline and 6 months in patients with and without ARDS

	ARDS (n = 26)	Non-ARDS (*n* = 41)	*P* value between-patient comparison^a^
BI baseline, mean ± SD	80.0 ± 24.7	87.8 ± 21.5	0.03
BI 6 months, mean ± SD	82.3 ± 22.9	89.6 ± 23.2	0.007
BI delta (difference in means)	2.3 (95 % CI –3.4, 8.0)	2.0 (95 % CI –3.2, 7.3)	0.5
*P* value within-patient comparison^b^	0.6	0.2	

^a^Wilcoxon rank sum test; ^b^Wilcoxon signed rank test. *ARDS* acute respiratory distress syndrome, *BI* Barthel index

Butterfly (stream) plots illustrate the trajectory of each patient’s functional status and QOL, showing no specific trend in either the ARDS or non-ARDS group (Additional file 2: Online Resources 2a, b and c).

Analysis of covariance was performed to evaluate whether ARDS itself was a predictor of QOL and BI at 6 months. After adjusting for age, sex and baseline value of SF-12 and BI, ARDS was not a statistically significant predictor of scores for the physical component of the SF-12 (*p* = 0.26), the mental component of SF-12 (*p* = 0.72), or the BI (*p* = 0.67).

Post-hoc sensitivity analysis was performed giving the lowest possible scores (zero) for the SF-12 and BI to the patients who were enrolled and died before 6-month follow up. There was no significant difference in the delta (baseline to 6 months) of SF-12 or BI between ARDS and non-ARDS groups (Additional file 3: Online Resource 3 and Additional file 4: Online Resource 4).

Post-hoc sensitivity analysis limited to self-answered surveys was performed as there was a difference in survey response (75 % of the non-ARDS group self-answered the survey, compared to 54 % of the ARDS group). The sensitivity analysis showed similar results, with no significant difference in the delta (baseline to 6 months) of the scores for SF-12 or BI between the ARDS and non-ARDS groups (Additional file 5: Online Resource 5 and Additional file 6: Online Resource 6).

## Discussion

In this prospective, population-based study of patients at risk of ARDS at the time of hospital admission, we observed no decline in functional status and QOL at 6 months compared to baseline, in patients who did and did not develop ARDS. Both groups had a lower baseline physical component score of the SF-12 compared to the US general population. Patients who developed ARDS had poor functional status compared to those who did not, both at baseline and at 6-month follow up.

An important finding of our study is that patients at risk of ARDS at hospital admission had significantly lower baseline QOL than the general population. Although critically ill survivors who developed ARDS required a longer duration of mechanical ventilation and ICU stay, they returned to their baseline QOL and functional status to the same extent as patients at risk who did not develop the syndrome. Previous studies showed that the post hospitalization QOL of ARDS survivors was lower than the US general population, especially the physical component score of SF-36, which is similar to our results. However, most of these studies did not have baseline QOL assessed [6, 8–10, 27], demonstrating only that ARDS survivors had a lower QOL than general population. Our data suggest that previously reported decline in QOL and functional status of ARDS survivors may at least in part be attributed to baseline disability, rather than being a consequence of critical illness and its treatment. Using different methodology, Iwashyna et al. [28] have also pointed out spurious inferences about long-term outcomes after critical illness, from studies that do not take into account the baseline status and trajectories of chronic conditions, particularly in the elderly. These findings may have important implications for designing the best strategies for treatment and prevention of long-term disability in survivors of critical illness.

In this study, patients who survived critical illness, regardless of whether they developed ARDS or not, returned to their pre-morbid QOL and functional status by 6 months. A few other studies with baseline assessment in a general ICU population have found similar results [29–35]. They found that QOL assessed many months after ICU discharge through the SF-36 in five of these studies [30–34] and through the Karnofsky performance status scores in one study [29], either returned to the baseline value or improved over time. Recently, a secondary analysis in a multicenter study have found pre-hospitalization QOL to be the main determinant of post-discharge QOL with minimal decrement due to hospitalization that was similar in ICU and non-ICU patients [36]. Functional decline attributed to critical illness noted in some of the previous studies [37, 38] may be related to different patient populations, different practice patterns and the timing of the assessment of baseline functional status.

An important difference between our study and the previous studies that prospectively evaluated QOL in ARDS survivors [6, 8] is the larger potential for selection bias inherent in clinical trials or referral center studies compared to population-based studies. For example, the average age of patients in our population-based study was much older than in the previous studies [6, 8]. Moreover, the former two studies were done almost ten years before our study, so changes in medical practice could have accounted for some of the discrepancies.

The main strengths of our study include the assessment of the baseline condition of the subjects and the comparison with a control group of patients at risk of ARDS who did not develop the syndrome. Very few studies have a control group of patients who are also critically ill but do not have ARDS [11, 39–41]. With the exception of one study [11], the other three studies [39–41] showed there was no significant difference in long-term QOL between patients with ARDS and critically ill patients without ARDS.

Our study has several limitations, starting with recruitment difficulties secondary to the decrease in ARDS incidence in Olmsted County, Minnesota [42], our sample size was smaller than intended; this not only affected the power of the study, but also it limited our ability to adjust for additional confounders. The study was stopped when preliminary analyses after three years showed a decreasing number of ARDS cases and no trend towards clinically significant differences between patients at risk who did and did not develop the syndrome. Therefore, the study was underpowered to evaluate small differences in QOL and functional status. However, clinically important differences are not likely to be missed as the effect sizes within groups and between groups were smaller than the minimal clinically important difference (MCID) of the SF-12 (0.5 of the SD = 5 points) [43]. Estimation of pre-morbid QOL was done by surrogates rather than patients in a significant number of ARDS patients, potentially affecting the accuracy of baseline QOL assessment [44].

Although population-based studies tend to have less selection bias than studies that follow patients from clinical trials, only 25 % of eligible patients at risk had baseline surveys completed in our study (Fig. 1), raising the issue of informative censoring. Although patients who were eligible but not enrolled had similar ARDS status and Charlson comorbidity index, they were older, more often treated in the ICU, had higher APACHE III score and had higher mortality than those enrolled. A number of patients who refused to participate did so due to change in goals of care which indeed might reflect poor prognosis. Also, some patients who completed the baseline survey died prior to completion of the follow-up survey at 6 months. This might represent a survival bias; however, this has been an issue throughout the studies involving long-term outcomes of critically ill patients. To address this issue, a post-hoc sensitivity analysis was performed and there was no significant difference between the two groups in the delta (baseline to 6 months) of SF-12 and BI scores. Finally, this is a single-center investigation with a largely Caucasian population, which limits the generalizability.

## Conclusions

In this prospective population-based study, survivors who did and did not develop ARDS returned to baseline QOL and functional status 6 months after hospitalization, though the physical component score of SF-12 remained lower than the average general US population in both groups. Our study results suggest that lower QOL and functional status after hospitalization were largely explained by baseline conditions, with similar recovery in survivors who did and did not develop ARDS.

## Key message

- In this prospective, population-based study of patients at risk of ARDS at the time of hospital admission, decreased quality of life and functional status 6 months after hospitalization were largely explained by their baseline condition, with similar recovery in survivors who did and did not develop ARDS

## Additional files

Additional file 1: Online Resource 1. Characteristics of eligible patients enrolled and not enrolled. (DOCX 17 kb) Additional file 2: Online Resource 2.
**a** Butterfly (stream) plot illustrates the changes in the mental component score of the 12-item Short Form survey (SF-12) of each individual patient from baseline (*middle line*) to 6 months follow up (*lateral line*). **b** Butterfly (stream) plot illustrates the changes in the physical component score of the SF-12 of each individual patient from baseline (*middle line*) to 6 months follow up (*lateral line*). c. Butterfly (stream) plots illustrate the changes in Barthel index score of each individual patient from baseline (*middle line*) to 6 months follow up (*lateral lines*). (ZIP 264 kb) Additional file 3: Online Resource 3. Mental and physical component score of the 12-item Short Form Survey (SF-12) at 6 months among groups of patients with and without acute respiratory distress syndrome (ARDS): sensitivity analysis with the lowest possible scores (zero) for those who died between baseline and their 6-month follow up. (DOCX 14 kb) Additional file 4: Online Resource 4. Barthel index (BI) score at baseline and 6 months among groups of patients with and without acute respiratory distress syndrome (ARDS): sensitivity analysis with the lowest possible scores (zero) for those who died between baseline and their 6 month follow up. (DOCX 15 kb) Additional file 5: Online Resource 5. Self-assessed mental and physical component score of 12-item Short Form Survey (SF-12) at baseline and 6 months groups of patients with and without acute respiratory distress syndrome (ARDS): sensitivity analysis limited to self-answered surveys. (DOCX 16 kb) Additional file 6: Online Resource 6. Self-reported Barthel index (BI) scores at baseline and 6 months among groups of patients with and without acute respiratory distress syndrome (ARDS): sensitivity analysis limited to self-answered surveys. (DOCX 15 kb)

## Abbreviations

ALI: acute lung injury
APACHE III: acute physiology and chronic health evaluation
ARDS: acute respiratory distress syndrome
BI: Barthel index
CONSORT: Consolidated Standards of Reporting Trials
HRQOL: health-related quality of life
ICU: intensive care unit
LIPS: lung injury prediction score
MCS: mental component score
MMSE: mini-mental status examination
MV: mechanical ventilation
PCS: physical component score
QOL: quality of life
SF-12: 12-Item Short Form Survey
SF-36: 36-Item Short Form Survey
