# Supplementary material for: Six-month quality-of-life and functional status of acute respiratory distress syndrome survivors compared to patients at risk: a population-based study
Source: Crit Care. 2015 Oct 2;19:356. doi: 10.1186/s13054-015-1062-y (PMC4591714; doi:10.1186/s13054-015-1062-y)
Supplement: Additional file 1: Online Resource 1. — Characteristics of eligible patients enrolled and not enrolled. (DOCX 17 kb) [file 13054_2015_1062_MOESM1_ESM.docx]

**Online Resource 1.** Characteristics of eligible patients enrolled and not enrolled

|  | Enrolled patients (n=98) | Eligible patients not enrolled (n=243)^*^ | P value |
| --- | --- | --- | --- |
| Age – median (IQR) | 62 (48.1, 72) | 67 (50, 79) | 0.04^1^ |
| Female sex – no. (%) | 44 (45) | 105 (43) | 0.78^2^ |
| White race – no. (%) | 89 (91) | 215 (88) | 0.53^2^ |
| ARDS during hospitalization – no. (%) | 41 (42) | 76 (31) | 0.06^2^ |
| Charlson Comorbidity Index - median (IQR) | 2 (1, 4) | 2 (1, 5) | 0.74^1^ |
| ICU admission – no. (%) | 53 (54) | 166 (68) | 0.01^2^ |
| Apache III score at 24 hours of ICU admission – median (IQR) | 71 (52, 80.5) | 80 (64, 104) | 0.0006^1^ |
| Mortality at 1 year – no. (%) | 25 (26) | 135 (56) | <0.0001^2^ |

Abbreviations: ARDS, acute respiratory distress syndrome; IQR, interquartile range.

^1^Wilcoxon rank sum test

^2^Chi square text

^*^ Patients who refused research authorization (58), patients whom ARDS status could not be determined (9) and duplicate patients (2) were excluded from this analysis
