# Supplementary figures and images for: Six-month quality-of-life and functional status of acute respiratory distress syndrome survivors compared to patients at risk: a population-based study
Source: Crit Care. 2015 Oct 2;19:356. doi: 10.1186/s13054-015-1062-y (PMC4591714; doi:10.1186/s13054-015-1062-y)

# SF12 Mental Component by ARDS

Bug Plot of Actual Values

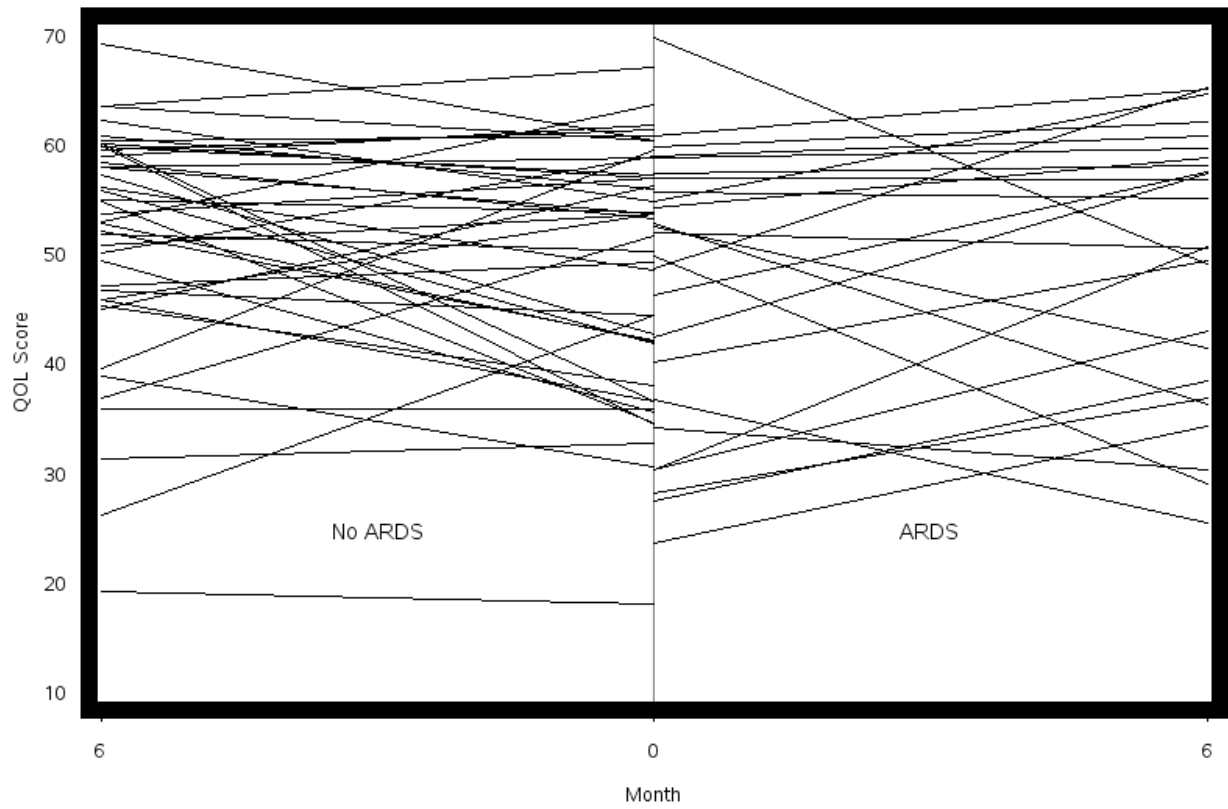

Supplement: Additional file 2: Online Resource 2. — a Butterfly (stream) plot illustrates the changes in the mental component score of the 12-item Short Form survey (SF-12) of each individual patient from baseline (middle line) to 6 months follow up (lateral line). b Butterfly (stream) plot illustrates the changes in the physical component score of the SF-12 of each individual patient from baseline (middle line) to 6 months follow up (lateral line). c. Butterfly (stream) plots illustrate the changes in Barthel index score of each individual patient from baseline (middle line) to 6 months follow up (lateral lines). (ZIP 264 kb) [file 13054_2015_1062_MOESM2_ESM.zip › OR_2a.pdf]

# SF12 Physical Component by ARDS

Bug Plot of Actual Values

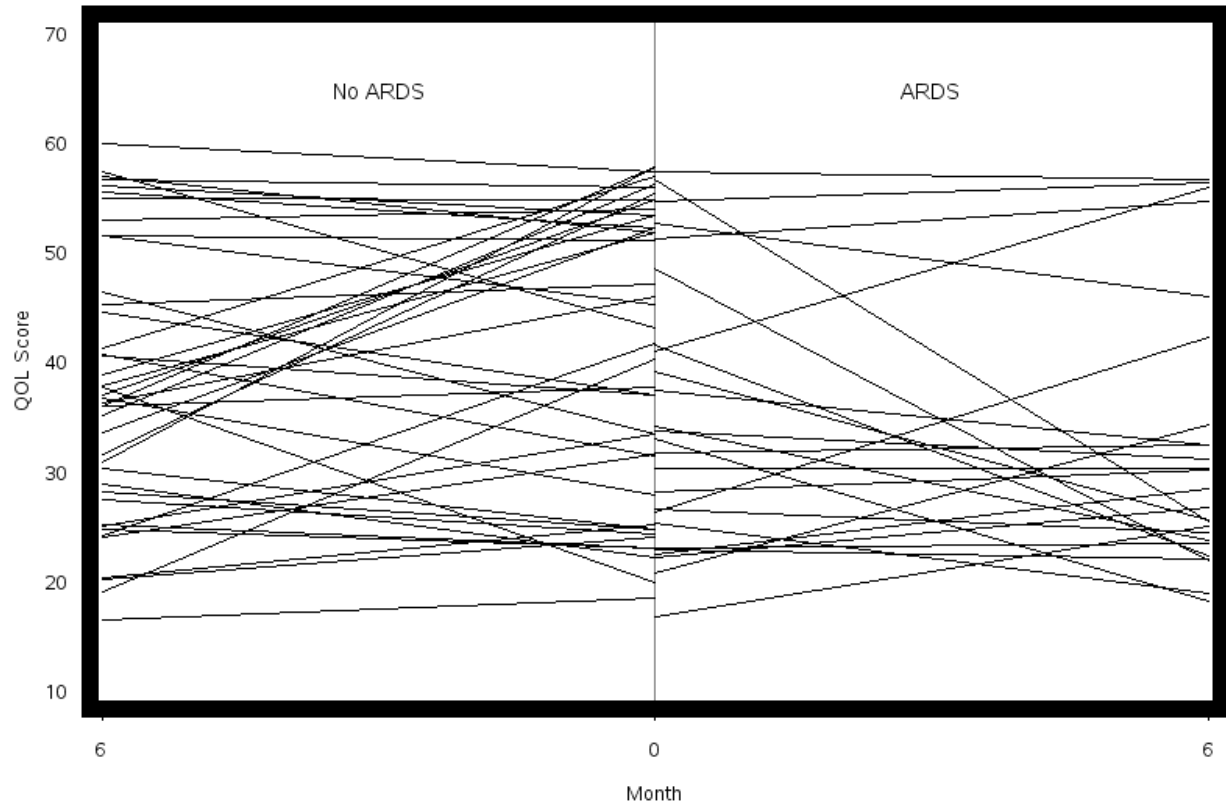

Supplement: Additional file 2: Online Resource 2. — a Butterfly (stream) plot illustrates the changes in the mental component score of the 12-item Short Form survey (SF-12) of each individual patient from baseline (middle line) to 6 months follow up (lateral line). b Butterfly (stream) plot illustrates the changes in the physical component score of the SF-12 of each individual patient from baseline (middle line) to 6 months follow up (lateral line). c. Butterfly (stream) plots illustrate the changes in Barthel index score of each individual patient from baseline (middle line) to 6 months follow up (lateral lines). (ZIP 264 kb) [file 13054_2015_1062_MOESM2_ESM.zip › OR_2b.pdf]

# Barthel Index by ARDS

Bug Plot of Actual Values

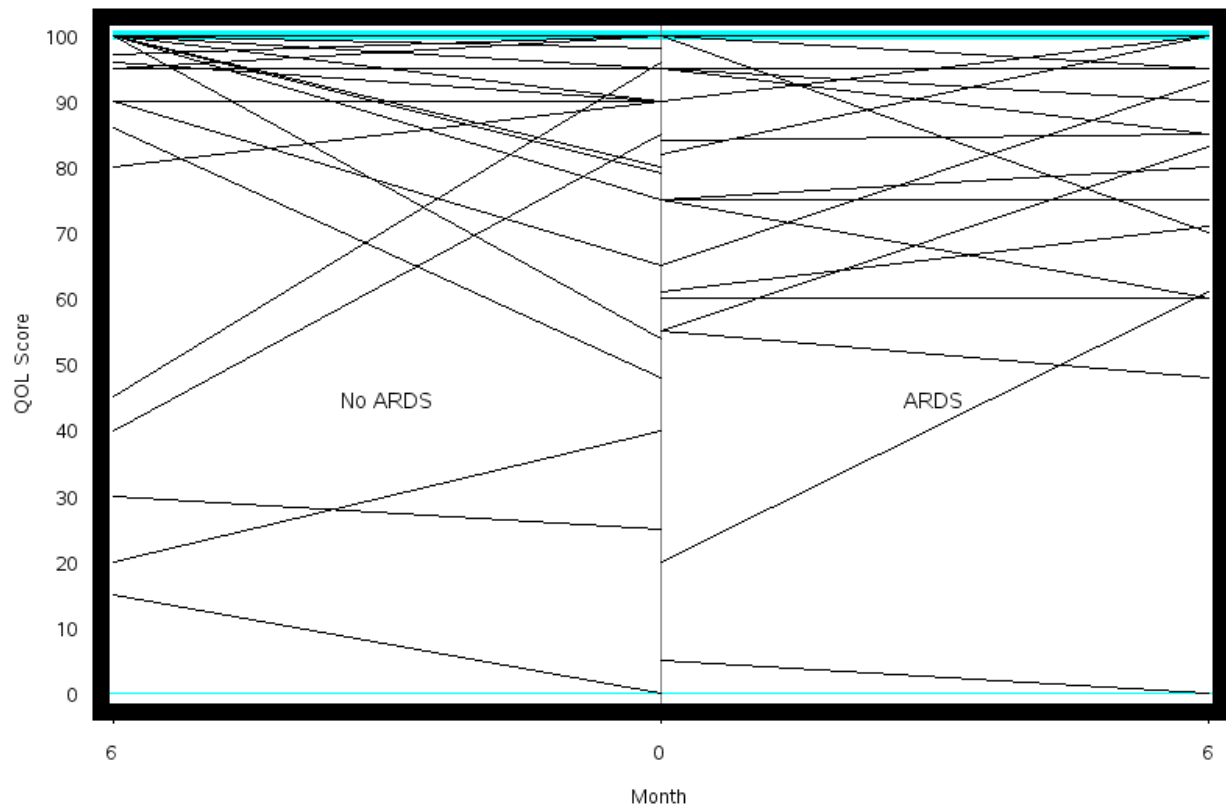

Supplement: Additional file 2: Online Resource 2. — a Butterfly (stream) plot illustrates the changes in the mental component score of the 12-item Short Form survey (SF-12) of each individual patient from baseline (middle line) to 6 months follow up (lateral line). b Butterfly (stream) plot illustrates the changes in the physical component score of the SF-12 of each individual patient from baseline (middle line) to 6 months follow up (lateral line). c. Butterfly (stream) plots illustrate the changes in Barthel index score of each individual patient from baseline (middle line) to 6 months follow up (lateral lines). (ZIP 264 kb) [file 13054_2015_1062_MOESM2_ESM.zip › OR_2c.pdf]
