# Supplementary material for: Six-month quality-of-life and functional status of acute respiratory distress syndrome survivors compared to patients at risk: a population-based study
Source: Crit Care. 2015 Oct 2;19:356. doi: 10.1186/s13054-015-1062-y (PMC4591714; doi:10.1186/s13054-015-1062-y)
Supplement: Additional file 3: Online Resource 3. — Mental and physical component score of the 12-item Short Form Survey (SF-12) at 6 months among groups of patients with and without acute respiratory distress syndrome (ARDS): sensitivity analysis with the lowest possible scores (zero) for those who died between baseline and their 6-month follow up. (DOCX 14 kb) [file 13054_2015_1062_MOESM3_ESM.docx]

**Online Resource 3.** Mental and physical component score of SF-12 at 6 months among ARDS and non-ARDS groups - sensitivity analysis with the lowest possible scores (zero) for those who died between baseline and their 6-month follow-up

|  | Non-ARDS (n=52) | ARDS (n=40) | P value^1^ |
| --- | --- | --- | --- |
| SF-12 MCS Baseline – mean (SD)^*^ | 48.7 (11.1) | 46.1 (12.4) | 0.3 |
| SF-12 MCS 6 months - mean (SD) | 40.5 (23.2) | 32.3 (25.9) | 0.1 |
| SF-12 MCS Delta (Baseline to 6 months) - mean (SD) | -7.8 (22.5) | -13.9 (24.8) | 0.2 |
| SF-12 PCS Baseline – mean (SD)^*^ | 41.2 (12.8) | 35.9 (12.5) | 0.045 |
| SF12 PCS 6 months - mean (SD) | 29.8 (19.0) | 21.3 (18.6) | 0.034 |
| SF12 PCS Delta (Baseline to 6 months) - mean (SD) | -10.1 (17.1) | -14.4 (20.1) | 0.3 |

Abbreviations: ARDS, acute respiratory distress syndrome; MCS, Mental Component Score; PCS, Physical Component Score; SD, standard deviation; SF-12, 12-Item Short Form Survey

^1^Independent *t* test

^*^Non-ARDS n=57, ARDS n=41
