# Supplementary material for: Six-month quality-of-life and functional status of acute respiratory distress syndrome survivors compared to patients at risk: a population-based study
Source: Crit Care. 2015 Oct 2;19:356. doi: 10.1186/s13054-015-1062-y (PMC4591714; doi:10.1186/s13054-015-1062-y)
Supplement: Additional file 4: Online Resource 4. — Barthel index (BI) score at baseline and 6 months among groups of patients with and without acute respiratory distress syndrome (ARDS): sensitivity analysis with the lowest possible scores (zero) for those who died between baseline and their 6 month follow up. (DOCX 15 kb) [file 13054_2015_1062_MOESM4_ESM.docx]

**Online Resource 4.**  BI at baseline and 6 months among ARDS and non-ARDS Groups - sensitivity analysis with the lowest possible scores (zero) for those who died between baseline and their 6 months follow-up

|  | Non-ARDS (n=52) | ARDS (n=40) | P value^1^ |
| --- | --- | --- | --- |
| BI Baseline – mean (SD)^*^ | 87.8 (21.5) | 80.0 (24.7) | 0.098 |
| BI 6 months - mean (SD) | 70.7 (42.3) | 53.5 (43.8) | 0.06 |
| BI Delta (Baseline to 6 months) - mean (SD) | -16.0 (39.1) | -26.0 (43.0) | 0.25 |

Abbreviations: ARDS, acute respiratory distress syndrome; BI, Barthel Index; SD, standard deviation

^1^Independent *t* test

^*^Non-ARDS n=57, ARDS n=41
