# Supplementary material for: Six-month quality-of-life and functional status of acute respiratory distress syndrome survivors compared to patients at risk: a population-based study
Source: Crit Care. 2015 Oct 2;19:356. doi: 10.1186/s13054-015-1062-y (PMC4591714; doi:10.1186/s13054-015-1062-y)
Supplement: Additional file 5: Online Resource 5. — Self-assessed mental and physical component score of 12-item Short Form Survey (SF-12) at baseline and 6 months groups of patients with and without acute respiratory distress syndrome (ARDS): sensitivity analysis limited to self-answered surveys. (DOCX 16 kb) [file 13054_2015_1062_MOESM5_ESM.docx]

**Online Resource 5** Self-Assessed Mental and Physical Component Score of SF-12 at Baseline and 6 Months among ARDS and Non-ARDS Groups - sensitivity analysis limited to self-answered surveys

|  | ARDS (n=15) | Non-ARDS (n=29) | P value for between-patient comparison^1^ |
| --- | --- | --- | --- |
| SF-12 MCS Baseline - mean±SD | 42.9±13.7 | 51.4±9.4 | 0.02 |
| SF-12 MCS 6 months - mean±SD | 44.7±14.3 | 53.1±8.8 | 0.02 |
| SF-12 MCS Delta (difference in means) | 1.8 (95% CI -5.6, 9.2) | 1.6 (95% CI -2.2, 5.5) | 0.96 |
| P value for within-patient comparison^2^ | 0.61 | 0.39 |  |
| SF-12 PCS Baseline - mean±SD | 31.6±10.5 | 43.7±13.5 | 0.02 |
| SF-12 PCS 6 months - mean±SD | 30.4±11.7 | 40.5.1±12.6 | 0.01 |
| SF-12 PCS Delta (difference in means) | -1.2 (95% CI -8.3, 5.9) | -3.3 (95% CI -7.6, 1.1) | 0.59 |
| P value for within-patient comparison^2^ | 0.72 | 0.14 |  |

Abbreviations: ARDS, acute respiratory distress syndrome; CI, confidence interval; MCS, Mental Component Score; PCS, Physical Component Score; SD, standard deviation; SF-12, 12-Item Short Form Survey

^1^Independent *t* test

^2^Paired *t* test
