# Supplementary material for: Six-month quality-of-life and functional status of acute respiratory distress syndrome survivors compared to patients at risk: a population-based study
Source: Crit Care. 2015 Oct 2;19:356. doi: 10.1186/s13054-015-1062-y (PMC4591714; doi:10.1186/s13054-015-1062-y)
Supplement: Additional file 6: Online Resource 6. — Self-reported Barthel index (BI) scores at baseline and 6 months among groups of patients with and without acute respiratory distress syndrome (ARDS): sensitivity analysis limited to self-answered surveys. (DOCX 15 kb) [file 13054_2015_1062_MOESM6_ESM.docx]

**Online Resource 6** Self Reporting BI Scores at Baseline and 6 Months among ARDS and Non-ARDS Groups - sensitivity analysis limited to self-answered surveys

|  | ARDS (n=14) | Non-ARDS (n=27) | P value between-patient comparison^1^ |
| --- | --- | --- | --- |
| BI Baseline - mean±SD | 79.0±26.4 | 94.2±12.4 | 0.01 |
| BI 6 months - mean±SD | 78.3±28.7 | 97.7±4.9 | 0.003 |
| BI Delta (difference in means) | -0.7 (95% CI –8.7, 7.3) | 3.6 (95% CI -0.37, 7.6) | 0.26 |
| P value within-patient comparison^2^ | 0.84 | 0.09 |  |

Abbreviations: ARDS, acute respiratory distress syndrome; BI, Barthel Index; SD, standard deviation

^1^Wilcoxon Rank Sum test

^2^Wilcoxon Signed Rank test
